# A cytoplasmic long noncoding RNA LINC00470 as a new AKT activator to mediate glioblastoma cell autophagy

**DOI:** 10.1186/s13045-018-0619-z

**Published:** 2018-06-04

**Authors:** Changhong Liu, Yan Zhang, Xiaoling She, Li Fan, Peiyao Li, Jianbo Feng, Haijuan Fu, Qing Liu, Qiang Liu, Chunhua Zhao, Yingnan Sun, Minghua Wu

**Affiliations:** 1 Hunan Provincial Tumor Hospital and the Affiliated Tumor Hospital of Xiangya Medical School, Central South University, Changsha, 410006 Hunan China; 2 Cancer Research Institute, School of Basic Medical Science, Central South University, Changsha, 410078 Hunan China; 3 Key Laboratory of Carcinogenesis and Cancer Invasion, Ministry of Education, Changsha, 410078 Hunan China; 4 Key Laboratory of Carcinogenesis, Ministry of Health, Changsha, 410078 Hunan China; 5 Second Xiangya Hospital, Central South University, Changsha, 410011 Hunan China; 6 Department of Biochemistry, University of California, Riverside, CA 92521 USA; 7 Xiangya Hospital, Central South University, Changsha, 410008 Hunan China; 8 Third Xiangya Hospital, Central South University, Changsha, 410013 Hunan China

**Keywords:** LncRNA, AKT activation, Oncogene, GBM

## Abstract

**Background:**

Despite the overwhelming number of investigations on AKT, little is known about lncRNA on AKT regulation, especially in GBM cells.

**Methods:**

RNA-binding protein immunoprecipitation assay (RIP) and RNA pulldown were used to confirm the binding of LINC00470 and fused in sarcoma (FUS). Confocal imaging, co-immunoprecipitation (Co-IP) and GST pulldown assays were used to detect the interaction between FUS and AKT. EdU assay, CCK-8 assay, and intracranial xenograft assays were performed to demonstrate the effect of LINC00470 on the malignant phenotype of GBM cells. RT-qPCR and Western blotting were performed to test the effect of LINC00470 on AKT and pAKT.

**Results:**

In this study, we demonstrated that LINC00470 was a positive regulator for AKT activation in GBM. LINC00470 bound to FUS and AKT to form a ternary complex, anchoring FUS in the cytoplasm to increase AKT activity. Higher pAKT activated by LINC00470 inhibited ubiquitination of HK1, which affected glycolysis, and inhibited cell autophagy. Furthermore, higher LINC00470 expression was associated with GBM tumorigenesis and poor patient prognosis.

**Conclusions:**

Our findings revealed a noncanonical AKT activation signaling pathway, i.e., LINC00470 directly interacts with FUS, serving as an AKT activator to promote GBM progression. LINC00470 has an important referential significance to evaluate the prognosis of patients.

**Electronic supplementary material:**

The online version of this article (10.1186/s13045-018-0619-z) contains supplementary material, which is available to authorized users.

## Background

AKT is a serine/threonine kinase, also known as protein kinase B, which plays critical roles in diverse cellular processes such as proliferation, autophagy, metabolism, and survival [1–4]. Aberrant AKT activation causes a wide variety of disorders including diabetes, neurodegenerative syndromes, and various types of cancers. AKT is well established as the predominant PI3K effector in many cell types [5]. Many cancer genetic alterations deregulate cell signaling pathways and exert their oncogenic effects in part through the PI3K/AKT pathway [6, 7]. Hence, there is a particularly intimate relationship between the activation of the AKT signaling pathway and tumorigenesis. Activation of PI3K results in the phosphorylation of two key residues on AKT, i.e., Thr308 in the activation motif and Ser473 in a C-terminal hydrophobic motif [7, 8]. AKT can translocate from the plasma membrane to intracellular compartments, including the cytoplasm and nucleus where it phosphorylates substrates [9, 10]. Growth factors stimulate phosphorylated AKT to translocate from the cytoplasm to the nucleus [11] where AKT can be phosphorylated and activated [12]. For example, nuclear AKT phosphorylates members of the Foxo subfamily of forkhead transcription factors, promoting nuclear exclusion and thereby inhibiting the transcription of death genes [13, 14]. Evidence indicates that a number of positive regulators, including regulatory proteins (such as PI3K, PTEN, PDK1) [15–17], miRNAs (such as miRNA-7, miRNA-379, and miRNA-126) [18–21], and long noncoding RNAs (lncRNAs, such as LINK-A, lncRNA OIP5-AS1, and MALAT1), promote the overactivation of AKT signaling [22–24]. Until now, the underlying mechanism of AKT in the GBM was not fully understood despite many years of investigation.

Recent studies have revealed the regulatory potential of many lncRNAs involved in numerous physiological and pathological processes [25]. LncRNAs have regulatory roles in gene expression at both transcriptional and post-transcriptional levels in diverse cellular contexts and biological processes [26]. LncRNAs are responsible for nuclear structure integrity and can regulate the expression of either nearby genes (acting in *cis* in the nucleus) or genes elsewhere in cells (acting in *trans* in the nucleus or cytoplasm) by interacting with proteins, RNA, and DNA [27–29]. LncRNAs operate through distinct modes, such as signals, scaffolds for protein-protein interactions, molecular decoys, or guides, to target elements in the genome [30, 31]. In addition, new types of lncRNAs are likely to be discovered through integrated approaches. For example, sno-lncRNA can form a nuclear accumulation that is enriched in RNA-binding proteins [32].

LINC00470 (also known as C18orf2) is a long non-coding RNA located in chromosome band 18p11.32 between RP11-16P11 and RP11-732L14 [33, 34]. Its alternative splicing of seven exons generates four transcripts. Our previous data demonstrated that LINC00470 expression levels in astrocytoma were significantly higher than those in normal brain tissues [35]. However, the role of LINC00470 remains to be elucidated; in particular, it is not known whether lncRNAs are involved in the regulation of AKT activity in GBM.

In this study, we found that (1) LINC00470 is a positive regulator of AKT activation and it inhibited the nuclear translocation of phosphorylated AKT; (2) LINC00470 directly bound FUS and anchored FUS in the cytoplasm, resulting in FUS activation; (3) LINC00470 interacted with FUS and AKT to form a stable complex; and (4) LINC00470 decreased the ubiquitination of HK1, which affected glycolysis by positively regulating AKT activation in GBM tumorigenesis.

## Methods

### Primary tumor cell culture and cell lines

A primary tumor cell culture was performed as previously described [36]. Astrocytoma cell lines U251 and U87 were bought from cell banks of the Chinese Academy of Sciences (Shanghai, China). All astrocytoma cell lines were subjected to a short tandem repeat (STR) test. U251 and primary tumor cells were cultured in DMEM high-glucose medium with 10% FBS and a 1% antibiotic-antimycotic solution (Gibco, Grand Island, NY, USA), while U87 cells were cultured in MEM medium with 10% FBS and 1% antibiotic-antimycotic solution at 37 °C and 5% CO_2_.

### Antibodies and reagents

The following primary antibodies were used: AKT (rabbit, Proteintech, 10176-2-AP, WB1:1500, IP:1:250, RIP:1:100); FUS (rabbit, Abcam, ab23439, WB1:2000, IP1: 200, RIP1:100); phospho-Akt (Ser473) (rabbit, Cell Signaling, #4060, WB1:1500); phospho-Akt (Thr308) (rabbit, Cell Signaling, #13038, WB1:1500); hexokinase I (rabbit, Cell Signaling, #2024, WB1:1000); hexokinase II (rabbit, Cell Signaling, #2867, WB1:1000); Flag (mouse, Sigma-Aldrich, F1804, IP 1:200); GAPDH (mouse, Sangon, D190090, WB 1:5000); H3 (rabbit, Beyotime, AH433, WB 1:500); and p53 (mouse, Active Motif, 39739, WB 1:1000, RIP 1:150). MK-2206 2HCl (S1078) was purchased from Selleck.

### LncRNA, siRNAs, and transfection

Cell transfection was performed using Lipofectamine 3000 (Invitrogen-Life Technologies, Carlsbad, CA, USA) per the manufacturer’s instructions.

### RNA isolation and RT-qPCR

This procedure was carried out as previously described. The following primers were used: LINC00470: F: 5′-CGTAAGGTGACGAGGAGCTG-3′, R: 5′-GGGGAATGGCTTTTGGGTCA-3′; AKT: F: 5′-GAAGGACGGGAGCAGGC-3′, R: 5′-AAGGTGCGTTCGATGACAGT-3′; and GAPDH: F: 5′-AATGGGCAGCCGTTAGGAAA-3′, R: 5′-GCGCCCAATACGACCAAATC-3′.

### Western blotting

Details of Western blotting were previously described [37]. Cell lysates were prepared with GLB buffer (10 mM Tris-HCl, pH = 7.5; 10 mM NaCl; 0.5% Triton X-100; 10 mM EDTA) supplemented with protease inhibitor cocktail (Bimake, Houston, TX, USA, B14001) and phosphatase inhibitor (Bimake, B15001). Cytoplasmic and nuclear proteins were prepared with a Nuclear and Cytoplasmic Protein Extraction Kit (Beyotime, p0028). Thirty-microgram proteins were subjected to electrophoresis in different percentages of gels according to the molecular weight of the detected proteins.

### Co-immunoprecipitation assay

For the interaction of FUS and AKT, HEK293 cells were transfected with the indicated plasmids and extracted by the addition of lysis buffer. For the immunoprecipitation of endogenous FUS and AKT proteins extracted by the addition of lysis buffer, the soluble supernatants were incubated with the indicated antibodies for 1 h at 4 °C. The immunocomplexes were then precipitated with protein A-Sepharose CL-4B. The immunocomplexes were washed three times with lysis buffer, eluted by boiling in sample buffer for SDS-PAGE, and then subjected to immunoblot analysis.

### Pulldown assay

GST fusion proteins containing various deletions of FUS cytoplasmic domain or deletions of AKT were expressed in U251 cells with the pGEX-4T-2 vector and were purified. The lysate was incubated for 1 h with GST-tagged proteins and glutathione-Sepharose 4B beads. The beads were subsequently washed three times in the lysis buffer containing 1 mM EDTA and 0.5 mM DTT. Precipitates were separated by SDS-PAGE and detected by Western blotting analysis.

### Cell viability and EdU assays

This procedure was carried out as previously described [35].

### RNA-binding protein immunoprecipitation assay

Approximately 2 μg of the cell extract was mixed with agarose beads, which had already precipitated with the protein antibodies. Beads were washed briefly three times with GLB^+^ lysis, and the retrieved protein was detected by Western blotting. The co-precipitated RNAs were detected by RT-qPCR.

### Intracranial implantation mouse model

All animal experiments were approved by the Animal Care and Use Committee of Central South University. Mouse orthotopic xenograft model was performed as previously described [36]. Six-week SD mice were chosen. Injection of cyclophosphamide once every 2 days. One-centimeter incision was made on the midline, and a 1-mm burr hole was drilled at AP = + 1 mm and MR = − 3 mm from the bregma at the right hemisphere. Ten microliters of 10^7^ cell was infused into the brain at a depth of − 5 mm from the dura, at a speed of 1 μl/min.

### Statistical analysis

All experiments were analyzed with GraphPad Prism 5 (La Jolla, CA, USA). Differences between the different groups were tested using Student’s *t* test or one-way ANOVA. The relationships between the LINC00470 expression and clinic-pathological parameters were examined using the *χ*^2^ test. The expression of LINC00470 and patients’ survival time was analyzed by single factor and multiplicity factor analysis, and OS curves were calculated using the Kaplan-Meier method with the SPSS 15.0 program (SPSS Inc., Chicago, IL, USA). Data are expressed as the mean ± S.E.M. from at least three independent experiments. A probability value *P* < 0.05 was considered statistically significant.

## Results

### LINC00470 was a positive regulator of AKT activities

**Fig. 1:**
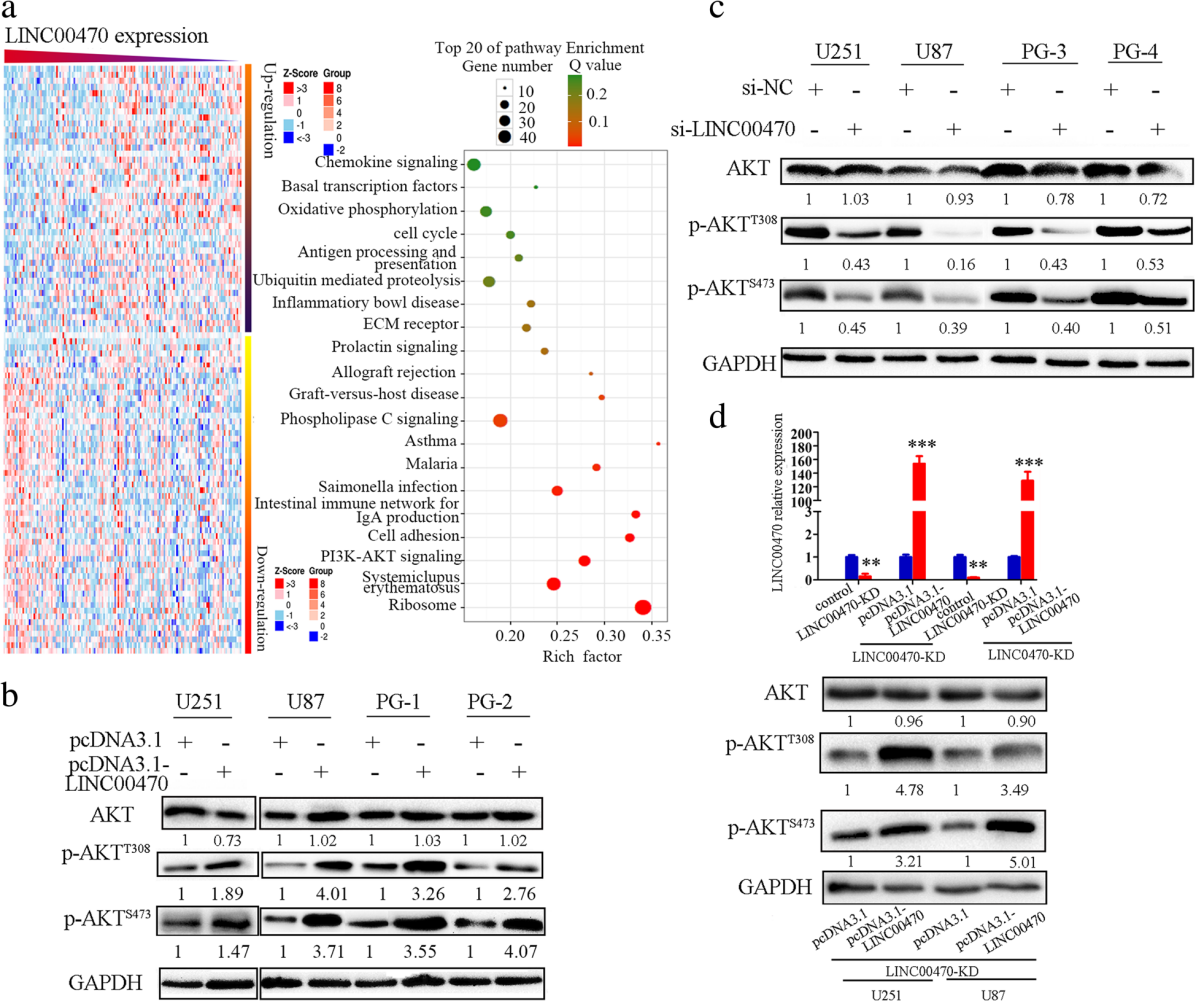
LINC00470 positively regulated the pAKT level. **a** Left, a heat map of LINC00470 correlated gene-expression signatures and the functional enrichment analysis of associated genes; right, enriched canonical pathways of the differentially expressed genes using Ingenuity Pathway Analysis (IPA). **b** Western blotting detected the expression levels of AKT, p-AKT^T308^, and p-AKT^S473^ in GBM cells by transfected them with pcDNA3.1- LINC00470. **c** Western blotting evaluated the expression levels of AKT, p-AKT^T308^, and p-AKT^S473^ in si-LINC00470-transfected GBM cells. **d** Upper, RT-qPCR measured the expression of LINC00470 in the LINC00470-KD GBM cell lines by re-expressing LINC00470; lower, Western blotting evaluated the expression levels of p-AKT^T308^ and p-AKT^S473^ and AKT in the LINC00470-KD GBM cells by re-expressing LINC00470. Data are presented as the mean ± S.E.M. of three independent experiments; **p < 0.01, ***p < 0.001

A vector construct containing the full-length LINC00470 with EGFP tag was developed and assessed for LINC00470 expression. LINC00470 did not have any detectable protein-coding ability (Additional file 1: Figure S1A and B). To investigate biological processes associated with LINC00470 expression in GBM, Pearson correlation analysis between LINC00470 expression and whole genome profiling were performed in GBM samples by TCGA databases. A total of 1802 gene expressions that correlated with LINC00470 expression are shown in Fig. 1a. To investigate which canonical pathways were significantly dysregulated in GBM groups with LINC00470 expression, Fisher’s exact test was used to identify 20 canonical pathways in GBM that included PI3K-AKT signaling (Fig. 1a). These analyses indicated that LINC00470 may be associated with PI3K-AKT signaling. Then, we measured the expression levels of LINC00470, AKT, and p-AKT in GBM cell lines and primary cultured GBM cells by RT-qPCR (Additional file 2: Figure S2A) and Western blotting (Additional file 2: Figure S2B). We found a positive correlation between the expression of LINC00470 and p-AKT. In GBM, LINC00470 and AKT had no correlation (Additional file 2: Figure S2B). Overexpression of LINC00470 upregulated the expression of p-AKT^T308^ and p-AKT^S473^ (Fig. 1b). We designed three kinds of siRNA and selected the best interfering effects for follow-up study (Additional file 3: Figure S3), while knockdown LINC00470 reduced p-AKT^T308^ and p-AKT^S473^ levels (Fig. 1c). We also re-expressed LINC00470 in the LINC00470-KD cells. Expression of LINC00470 was elevated after LINC00470 was re-expressed in the LINC00470-KD cells (Fig. 1d upper) and enhanced the p-AKT^T308^ and p-AKT^S473^ level (Fig. 1d lower panel). However, neither overexpression nor knockdown of LINC00470 affected total AKT and PI3K expression (Fig. 1b, c and Additional file 4: Figure S4). Therefore, we proposed that LINC00470 modulates AKT activities possibly through a previously unidentified mechanism.

### FUS interacted with both LINC00470 and AKT to form a ternary complex in the cytoplasm

**Fig. 2:**
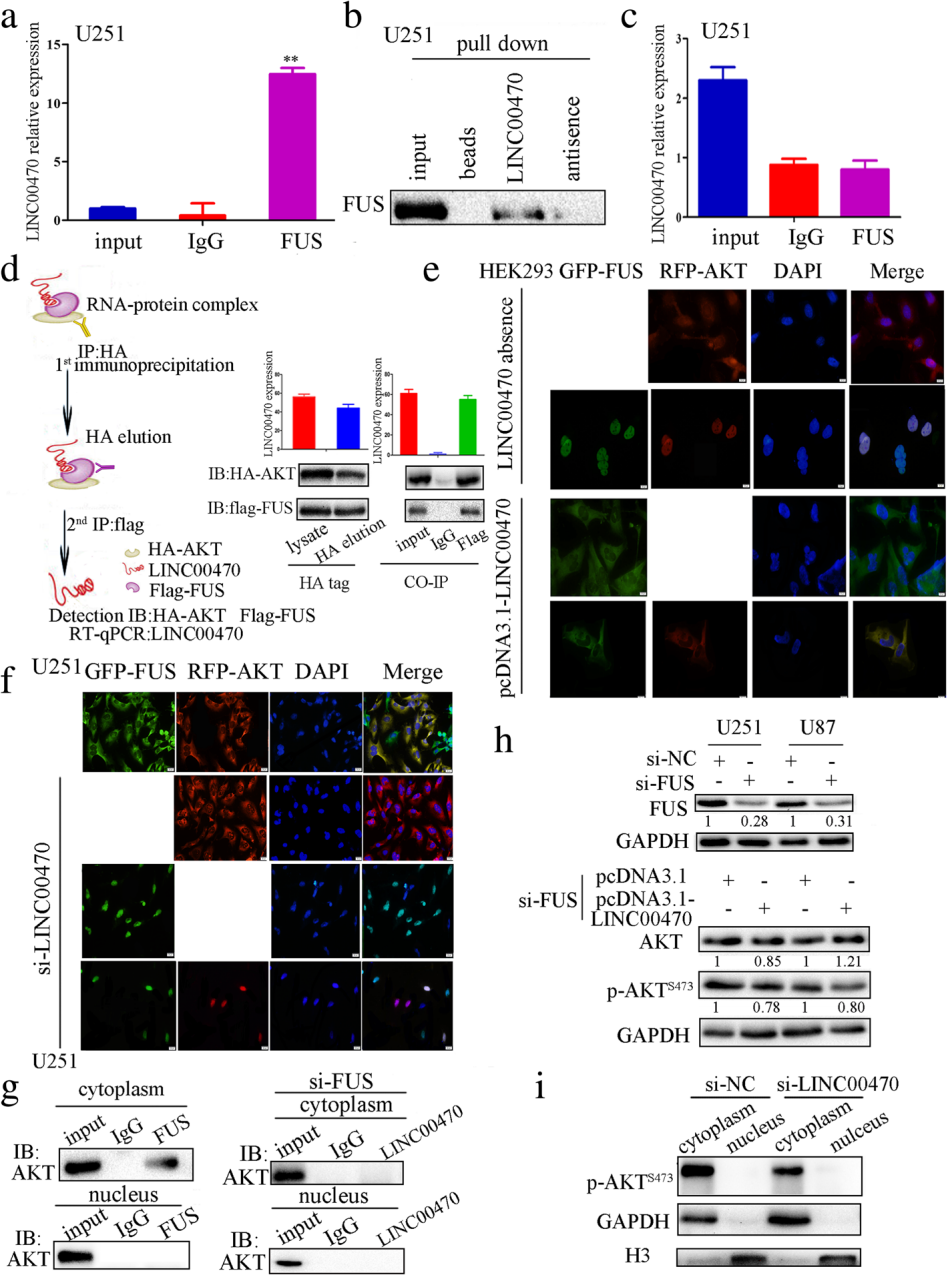
FUS interacted with LINC00470 and AKT to form a ternary complex in the cytoplasm. **a** The interaction of LINC00470 and FUS was detected through RIP assays in U251 cells. Data are presented as the mean ± S.E.M. of three independent experiments. **p < 0.01. **b** RNA pulldown showed binding between LINC00470 and FUS. **c** RIP assays showed that there was no interaction between LINC00470 and AKT in U251 cells. Data are presented as the mean ± S.E.M. of three independent experiments. **d** HEK293 cells were transfected with HA-AKT, Flag-FUS, and pcDNA3.1-LINC00470. Two-step co-immunoprecipitation verified their interaction. The expression levels of LINC00470, AKT, and FUS were measured with RT-qPCR and Western blotting, respectively. **e** The localization of AKT and FUS was detected by immunofluorescence staining in HEK293 cells. **f** The co-localization of AKT and FUS was detected by immunofluorescence staining in U251 cells. **g** Left, the interactions between endogenous FUS and AKT in the cytoplasm and nucleus were measured by co-immunoprecipitation; right, an RNA pulldown assay showed binding between endogenous LINC00470 and AKT in the cytoplasm and nucleus of U251 cells transfected by si-FUS. **h** Western blotting detected the expression of FUS in GBM cells transfected by si-FUS. Expression levels of AKT and p-AKT^S473^ were measured by Western blotting in GBM cells that re-expressed LINC00470 in FUS-KD GBM cells. **i** Western blotting detected the expression levels of p-AKT^S473^ in the cytoplasm and nucleus of U251 cells transfected by si-LINC00470

Bioinformatics (http://starbase.sysu.edu.cn/browseRbpLncRNA.php) predicted FUS, an RNA-binding protein associated with LINC00470, may also bind to AKT. An RIP assay verified the interaction between LINC00470 and FUS (Fig. 2a). A biotin-RNA pulldown assay further confirmed the binding between LINC00470 and FUS (Fig. 2b). Interestingly, RNA-binding protein immunoprecipitation of FUS, but not AKT, specifically retrieved LINC00470 (Fig. 2c). We also performed RIP assay in U87 cells; the result of RIP assay was consistent with that in U251cells (Additional file 5: Figure S5). These results indicated that FUS interacted with LINC00470, but there was no direct interaction between LINC00470 and AKT.

To examine the simultaneous existence of LINC00470, FUS, and AKT within the same complex, a two-step Co-IP assay was performed using HEK293 cell lysate from cells in which HA-AKT, Flag-FUS, and pcDNA3.1-LINC00470 were co-transfected. LINC00470 was found in the final immunoprecipitation, suggesting that LINC00470, FUS, and AKT form a ternary complex (Fig. 2d). At the same time, we also found LINC00470, FUS, and AKT can form a ternary complex in U251 cells (Additional file 6: Figure S6). In the absence of LINC00470, FUS and AKT were co-localized in the nucleus of HEK293 cells (Additional file 7: Figure S7 and Fig. 2e). However, overexpression of LINC00470 anchored FUS and AKT in the cytoplasm (Fig. 2e), while with knockdown of LINC00470 in U251 cells, FUS and AKT translocated from the cytoplasm to the nucleus (Fig. 2f). The interactions between FUS and AKT in the cytoplasm of U251 cells were also verified by Western blotting analysis (Fig. 2g left). An RNA pulldown assay showed that the interaction of LINC00470 and AKT disappeared after FUS knockdown in U251 cells (Fig. 2g right). We also found LINC00470 could not affect AKT activation after pcDNA3.1-LINC00470 was transfected into FUS-KD cells (Fig. 2h). The phosphorylated AKT was reduced in the cytoplasm in LINC00470-knockdown GBM cells (Fig. 2i). The data indicated that LINC00470 promoted the activation of AKT in the cytoplasm by interacting with FUS.

### LINC00470 anchored FUS in the cytoplasm and phosphorylated FUS

**Fig. 3:**
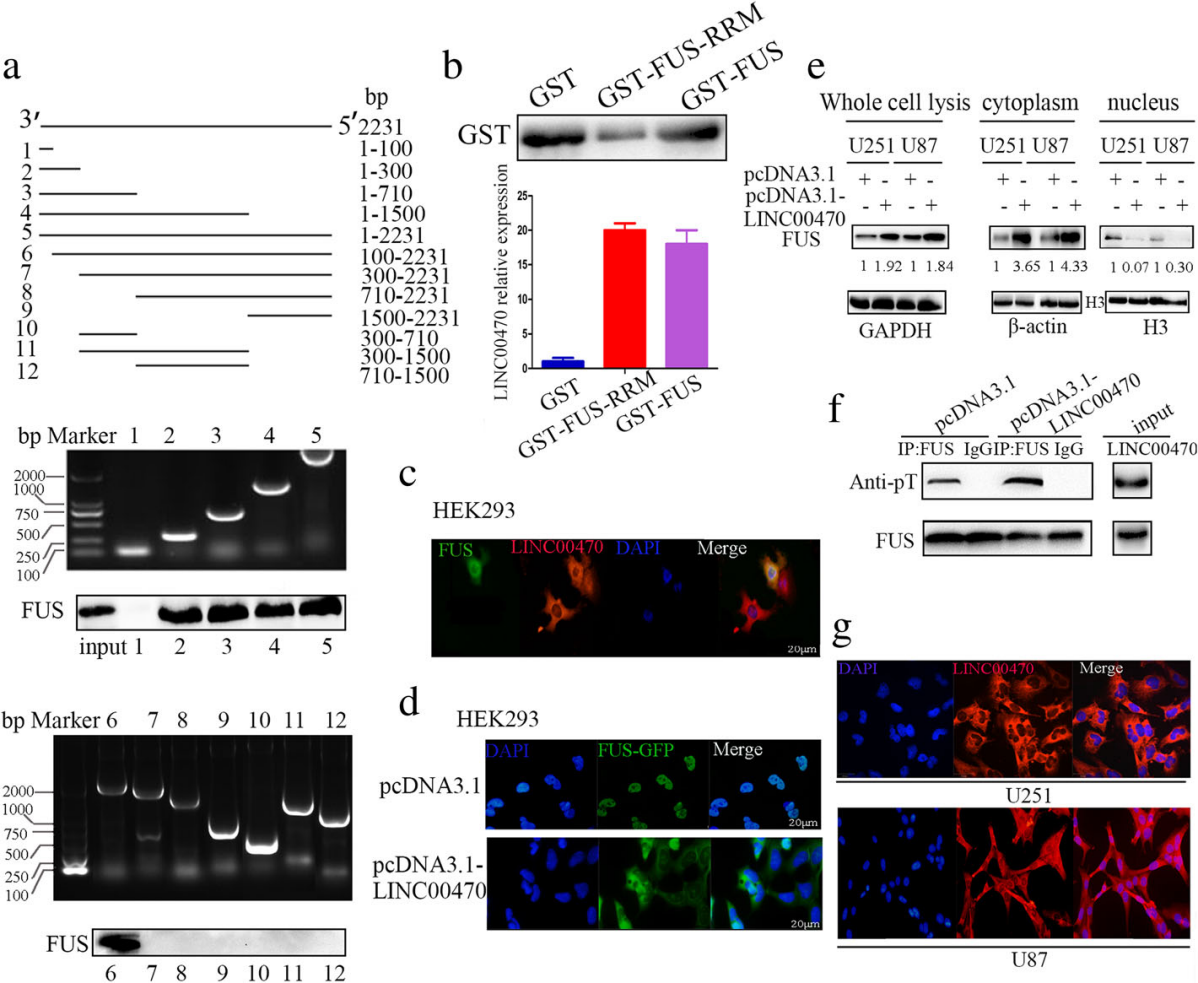
LINC00470 anchored FUS in the cytoplasm and phosphorylated FUS. **a** Upper, schematic illustration of substitution mutant constructs of LINC00470; middle and lower, an RNA pulldown assay examined the interaction between FUS and the different mutants of LINC00470. **b** GST pulldown assays showed that the RRM domain of FUS pulled down LINC00470. **c** Representative immunofluorescence staining displayed the co-localization of LINC00470 and FUS in the cytoplasm of HEK293 cells after LINC00470 overexpression. Scale bar, 20 μm. **d** Representative imaging of LINC00470 anchoring FUS in the cytoplasm in HEK293 cells. Scale bar, 20 μm. **e** Western blotting measured the expression of FUS in whole cell lysis and the cytoplasm and nucleus in U251 cells transfected by pcDNA3.1-LINC00470. **f** Representative immunoprecipitation analysis detected FUS phosphorylation in U251 cells transfected by pcDNA3.1-LINC00470. FUS immunoprecipitated from U251 cells was immunoblotted with pan-phospho-S/TQ antibodies to assess the phosphorylation level of FUS. **g** RNA fluorescence in situ hybridization showed the localization of LINC00470 in GBM cells. The nucleus was counterstained with DAPI. Scale bar, 29 μm

Next, a series of LINC00470 deletion mutants were constructed to determine the nucleotides in LINC00470 that bind to FUS. An RNA pulldown assay showed that there was an interaction between FUS and LINC00470 mutants (1–300 nt, 1–710 nt, 1–1500 nt, 1–2231 nt, and 100–2231 nt), but no interaction between FUS and other LINC00470 deletion mutants (1–100 nt, 300–2231 nt, 710–2231 nt, 1500–2231 nt, 300–710 nt, 300–1500 nt, and 710–1500 nt) (Fig. 3a), suggesting that the 100–300 nt region of LINC00470 was responsible for its binding to FUS. We also found FUS bound to LINC00470 through its RNA recognition domain (RRM) (Fig. 3b). Confocal fluorescence microscopy indicated that LINC00470 and FUS were mainly co-localized in the cytoplasm in HEK 293 cells after overexpression of LINC00470 (Fig. 3c).

FUS was reported to be continuously shuttling between the nucleus and the cytoplasm [38]. FUS contains multiple post-translational modification sites in the RRM and GGR domain, and post-translational modifications of FUS have profound effects on its binding capacity of DNA, RNA and proteins, changes in protein stability, or subcellular localization [39, 40]. We speculated that LINC00470 may impact FUS subcellular localization. The nuclear localization of FUS was explored by transient expression of the GFP-FUS fusion plasmid in HEK293 cells (Fig. 3d). When HEK293 cells were co-transfected by pcDNA3.1-LINC00470 and the GFP-FUS fusion plasmid, LINC00470 led to the translocation of FUS from the nucleus to the cytoplasm (Fig. 3d). In GBM cells, the expression of FUS was increased by LINC00470 overexpression and the FUS level was significantly increased in cytoplasm; however, its expression was decreased in the nucleus (Fig. 3e). These data suggested that LINC00470 anchored FUS in the cytoplasm and promoted its expression in the cytoplasm. FUS immunoprecipitation from U251 cells after overexpression of LINC00470 was immunoblotted with anti-phospho-T (threonine phosphorylation) antibodies to assess the level of FUS phosphorylation; LINC00470 promoted phosphorylation of FUS at threonine residues (Fig. 3f). In addition, we also found that LINC00470 was mainly located in the cytoplasm in GBM cells by RNA fluorescence in situ hybridization (Fig. 3g).

### FUS bound to AKT and promoted AKT nuclear translocation and activation

**Fig. 4:**
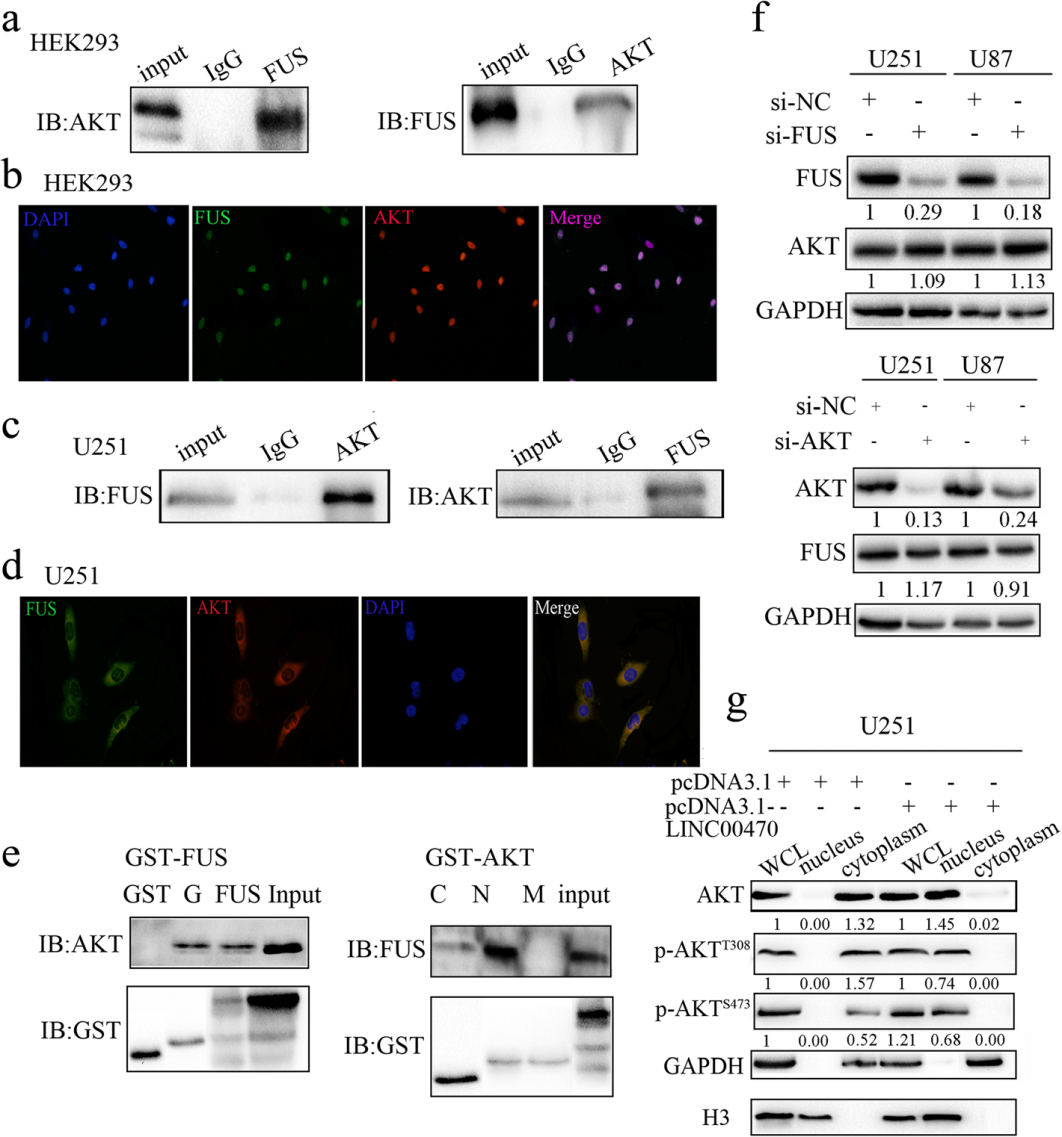
FUS bound to AKT and promoted AKT activation. **a** Co-IP analysis measured the exogenous interaction between FUS and AKT in HEK293 cells. **b** Representative immunofluorescence staining displayed the co-localization of FUS and AKT in the nucleus of HEK293 cells. **c** Co-IP analysis measured the endogenous interaction between FUS and AKT in U251 cells. **d** Representative immunofluorescence staining displayed the endogenous co-localization of FUS and AKT in the cytoplasm of U251 cells. **e** Left, GST pulldown assays showed that the GGR domain of FUS pulled down AKT; right, GST pulldown assays showed that the N-terminal region of AKT mainly pulled down FUS. **f** Upper, Western blotting measured the expression levels of FUS and AKT in GBM cells transfected by si-FUS; lower, Western blotting measured the expression levels of AKT and FUS in GBM cells transfected with si-AKT. **g** Western blotting measured the expression levels of AKT and pAKT in the whole lysis, cytoplasm, and nucleus of U251 cells transfected by pcDNA3.1-FUS

The “Scansite 2.0” software was utilized to identify a docking domain (GGR domain) in FUS, which is an AKT kinase-binding site. GFP-FUS and RFP-AKT expression plasmids were co-transfected into HEK293 cells, CO-IP and immunofluorescence suggested there were interactions between FUS and AKT, and both were co-localized in the nucleus of HEK293 cells (Fig. 4a, b). In addition, we confirmed that endogenous AKT interacted with FUS in the cytoplasm of U251 cells (Fig. 4c, d). Next, a fusion protein of the GGR domain mutation in FUS (GST-FUS-GGR domain) was constructed. A GST pulldown assay indicated that AKT was precipitated with the GST-FUS-GGR peptide (Fig. 4e left) and FUS mainly bound with the N domain of AKT (Fig. 4e right). Then, we analyzed the changes in AKT protein levels after silencing FUS. Knockdown of FUS did not affect AKT expression. Similarly, FUS expression was not affected by silencing AKT (Fig. 4f). However, we observed that FUS influenced the subcellular localization of AKT by promoting AKT nuclear translocation and increased AKT activation in the nucleus (Fig. 4g).

### LINC00470 decreased ubiquitination of HK1 to affect glycolysis by positively regulating AKT activation

**Fig. 5:**
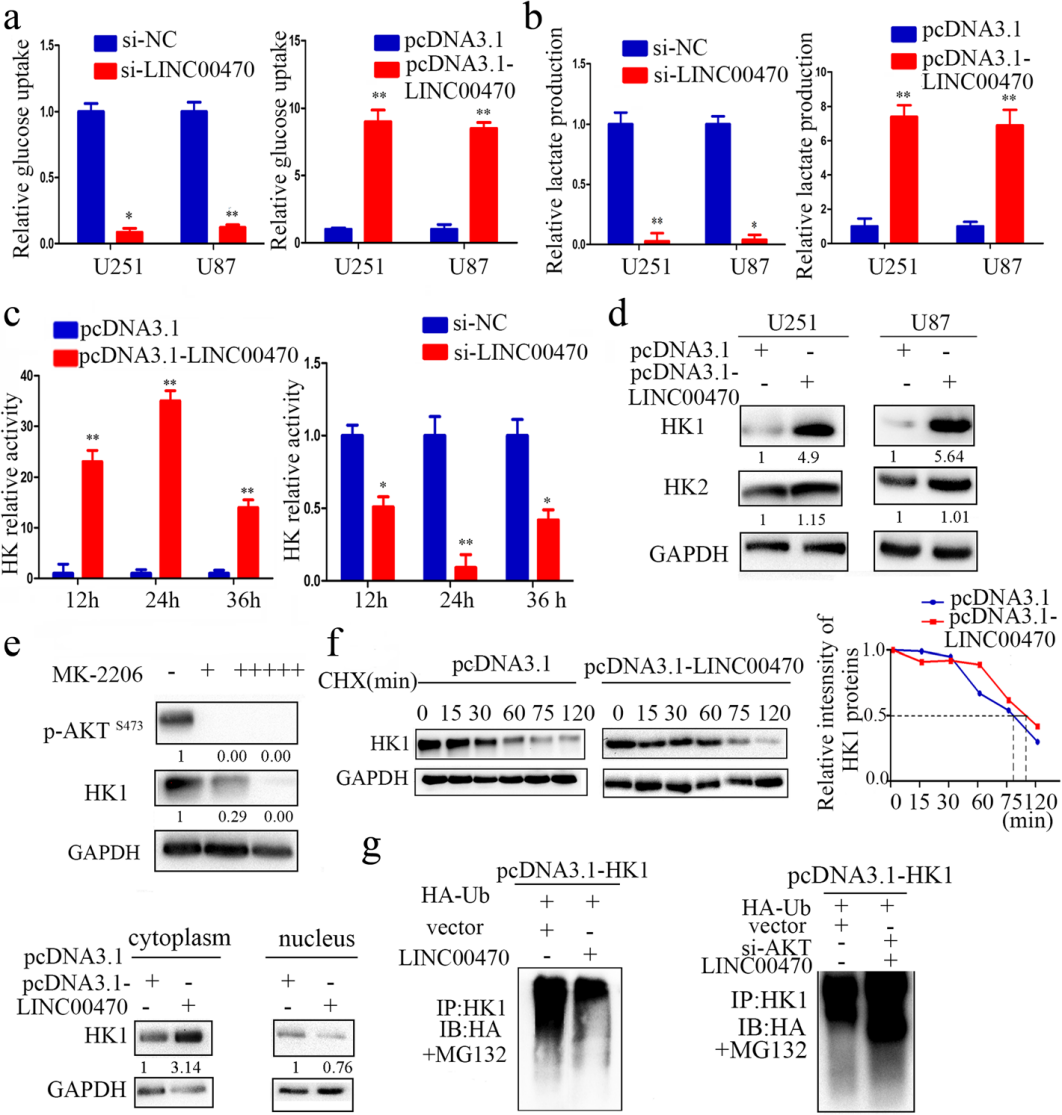
LINC00470 inhibited HK1 ubiquitination to affect glycolysis by positively regulating AKT activation. **a** RT-qPCR measured the expression of LINC00470 in the GBM cell lines; GBM cells were transfected with si-LINC00470 or pcDNA3.1-LINC00470. Data are presented as the mean ± S.E.M. of three independent experiments; *p < 0.05, **p < 0.01. **b** Relative levels of glucose uptake and lactate production were detected in GBM cells. GBM cells were transfected with si-LINC00470 or pcDNA3.1-LINC00470. Data are presented as the mean ± S.E.M. of three independent experiments; *p < 0.05, **p < 0.01. **c** HK activity was measured at different time points after GBM cells were transfected with pcDNA3.1-LINC00470 or si-LINC00470. Data are presented as the mean ± S.E.M. of three independent experiments; **p < 0.01, ***p < 0.001. **d** Western blotting detected the expression levels of HK1 and HK2 in GBM cells transfected by LINC00470. **e** Upper, Western blotting detected the expression levels of p-AKT^S473^ and HK1 in U251 cells. The cells were treated with different concentrations of AKT inhibitor (MK-2206; +, 1 μM; +++++, 5 μM); lower, Western blotting detected the expression levels of HK1 in the cytoplasm and nucleus in U251 cells transfected by pcDNA3.1-LINC00470. **f** The half-life of HK1 was assessed in U251 cells. Cells were transfected with pcDNA3.1-LINC00470. **g** The relative amount of ubiquitination HK1 was determined by a ubiquitination assay in U251 cells transfected by LINC00470 or si-AKT and LINC00470

AKT, which is frequently dysregulated in cancer, is a well-established regulator of glucose metabolism [41]. Its regulation on metabolic processes is required for tumor proliferation, apoptosis, and autophagy [42–44]. Enforcing or silencing LINC00470 expression in GBM cells increased or reduced glycolysis uptake and lactate production, respectively (Fig. 5a, b). Hexokinases catalyze the first and irreversible step of glucose metabolism, i.e., the ATP-dependent phosphorylation of glucose to yield glucose-6-phosphate [45]. Overexpression of LINC00470 increased the total hexokinase activity in U251 cells compared to controls, and HK activity was inhibited after knockdown of LINC00470 in U251 cells (Fig. 5c). HK1 is a major isoform of HK and is the first key enzyme in the glycolysis pathway [45]. Importantly, the protein expression level of HK1 was markedly increased in response to LINC00470 overexpression (Fig. 5d). In contrast, we found that HK2, another major isoform of HK, was not changed statistically significantly in LINC00470-overexpressed cells (Fig. 5d).

Next, we explored the molecular mechanisms underlying the LINC00470 that affects the activity of HK1. Inhibiting the activity of AKT with MK-2206 resulted in downregulated of HK1 (Fig. 5e). Additionally, we transfected both pcDNA3.1 and pcDNA3.1-LINC00470 vectors into U251 cells, then analyzed the protein levels of HK1 in the cytoplasm and nucleus. HK1 expression increased in the cytoplasm under different experimental conditions; concomitantly, HK1 expression in the nucleus did not change significantly (Fig. 5e). To determine how HK1 protein changed, we treated U251 cells with cycloheximide (CHX) and analyzed the stability of HK1 in response to LINC00470 overexpression. The half-life of HK1 was much longer in LINC00470-overexpressed cells than that in controls (Fig. 5f). We further explored the mechanism of AKT-mediated HK1 regulation and found lower HK1 ubiquitination levels in LINC00470-transfected cells treated with MG132, and a restoration experiment was performed by knocking down AKT. We found that the ubiquitination level of HK1 was rescued (Fig. 5g). Together, these observations suggested that LINC00470 affected the ubiquitination and expression of HK1 through activating AKT.

### LINC00470 is an onco-RNA, and it induced the malignant characteristics of GBM cells

**Fig. 6:**
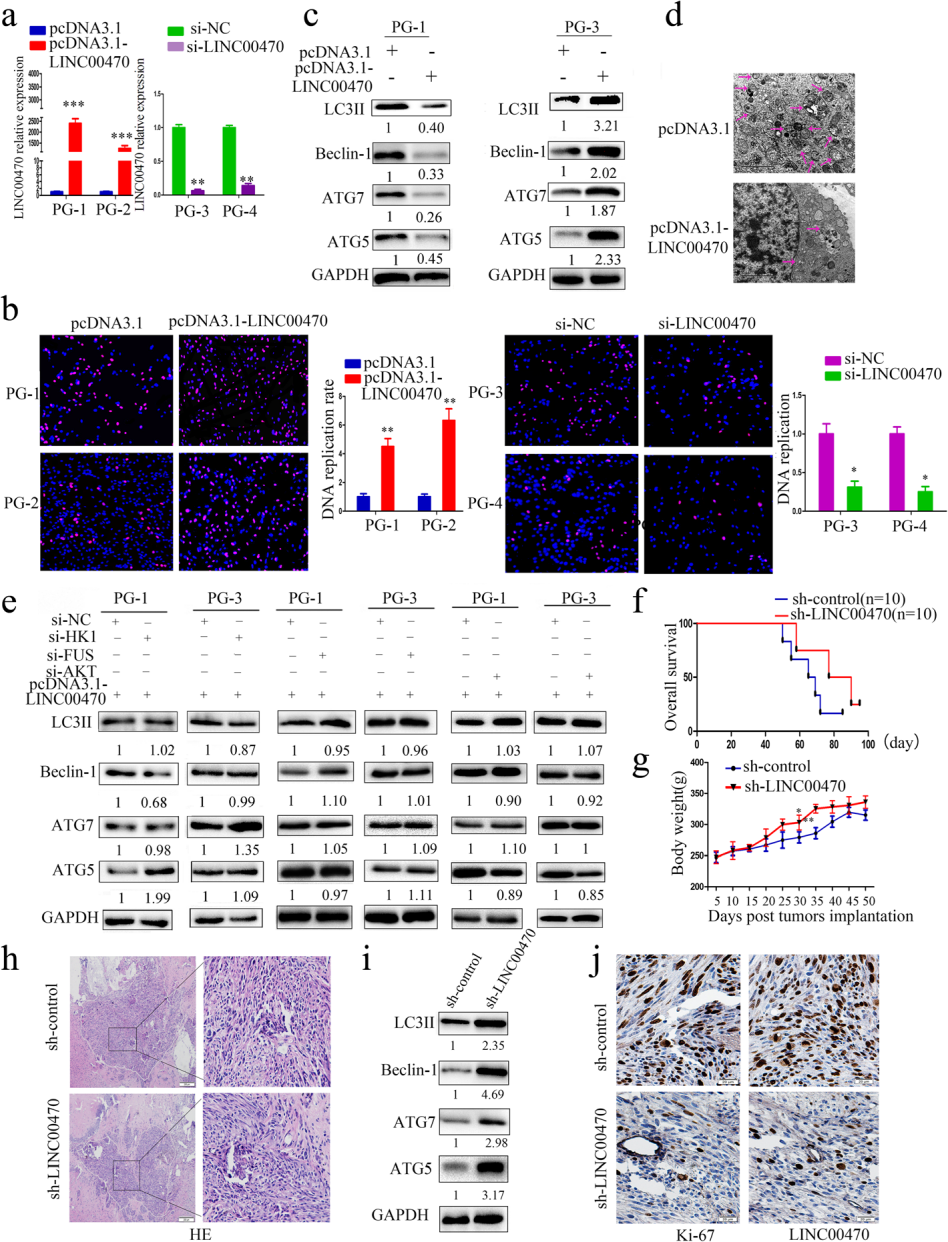
LINC00470 promoted the tumorigenesis of GBM cells. **a** Expression levels of LINC00470 were measured by RT-qPCR in primary cultured GBM cells (LINC00470 had relatively low expression in PG-1 and PG-2; LINC00470 had relatively high expression in PG-3 and PG-4). Primary cultured GBM cells were transfected with si-LINC00470 or pcDNA3.1-LINC00470. Data are presented as the mean ± S.E.M. of three independent experiments; **p < 0.01, ***p < 0.001. **b** An EDU assay was applied to assess cell proliferation of primary cultured GBM cells. Primary cultured GBM cells were transfected with pcDNA3.1-LINC00470 or si-LINC00470. **c** Western blotting measured the expression levels of autophagy marker LC3, beclin-1, ATG7, and ATG5 in PG-1 and PG-3 cells. The cells were transfected with pcDNA3.1-LINC00470 or si-LINC00470. **d** Electron microscopy detected the autophagy of U251 cells transfected with pcDNA3.1-LINC00470. **e** Western blotting measured the expression levels of autophagy marker LC3, beclin-1, ATG7, and ATG5 in PG-1 and PG-3 cells. The cells were transfected with si-HK1, si-FUS or si-AKT. **f** Survival analysis showed that Sprague Dawley rats transplanted with U251-sh-LINC00470 cells have longer overall survival. **g** Tumor growth for U251-sh-control and U251-sh-LINC00470 in Sprague Dawley rats.*p < 0.05, **p < 0.01. **h** H&E staining showed the volume and morphology of tumors in mice transplanted with U251-sh-LINC00470 cells. The white circle represents the size of the tumor. **i** Western blotting measured the expression levels of the autophagy marker LC3, beclin-1, ATG7, and ATG5 in intracranial transplanted tumors. **j** Expression of Ki-67 and LINC00470 in intracranial transplanted tumors was detected by immunohistochemical staining or in situ hybridization, respectively

The above data suggested that LINC00470 plays an important role in GBMs. Accordingly, the primary cultured cells were used to evaluate the functions of LINC00470. As shown in Additional file 7: Figure S7, there was relatively low expression of LINC00470 in PG-1 and PG-2 cells and relatively high expression of LINC00470 in PG-3 and PG-4 cells. Therefore, we expected overexpression of LINC00470 in PG-1 and PG-2 cells and knockdown of LINC00470 in PG-3 and PG-4 cells (Fig. 6a). We found overexpression of LINC00470 contributed to the proliferation of PG1 and PG2 cells by CCK-8 assay (Fig. 6b and Additional file 8: Figure S8). Knockdown of LINC00470 in PG-3 and PG-4 cells decreased cell proliferation (Fig. 6b and Additional file 8: Figure S8). Autophagy primarily promoted the progression of cancers [46, 47]. We also found that, in PG-1 cells, overexpression of LINC00470 inhibited the levels of autophagy (Fig. 6c, d), and in PG-3 cells, knockdown of LINC00470 promoted the levels of autophagy (Fig. 6c).

To evaluate whether glycolysis activation serves as an upstream mechanism for LINC00470-mediated autophagy, we monitored the markers of autophagy by knockdown of HK1, FUS, and AKT, respectively. The results showed that when HK1, FUS, or LINC00470 was knocked down, the autophagy level of GBM did not decrease. These results suggested that LINC00470 affected autophagy that was required for HK1, FUS, and AKT (Fig. 6e). At the same time, we applied an intracranial orthotopic transplanted model to evaluate whether LINC00470 mediated GBM tumorigenesis. Compared to mice transplanted with U251-sh-control cells, mice transplanted with U251-sh-LINC00470 cells exhibited longer survival (Fig. 6f), gained more weight (Fig. 6g), and had smaller tumors (Fig. 6h). Knockdown of LINC00470 significantly increased the autophagy levels and decreased the expression of Ki-67 and LINC00470 in an intracranial orthotopic transplanted model (Fig. 6i, j).

### LINC00470 was an independent prognostic factor in astrocytoma patients

**Fig. 7:**
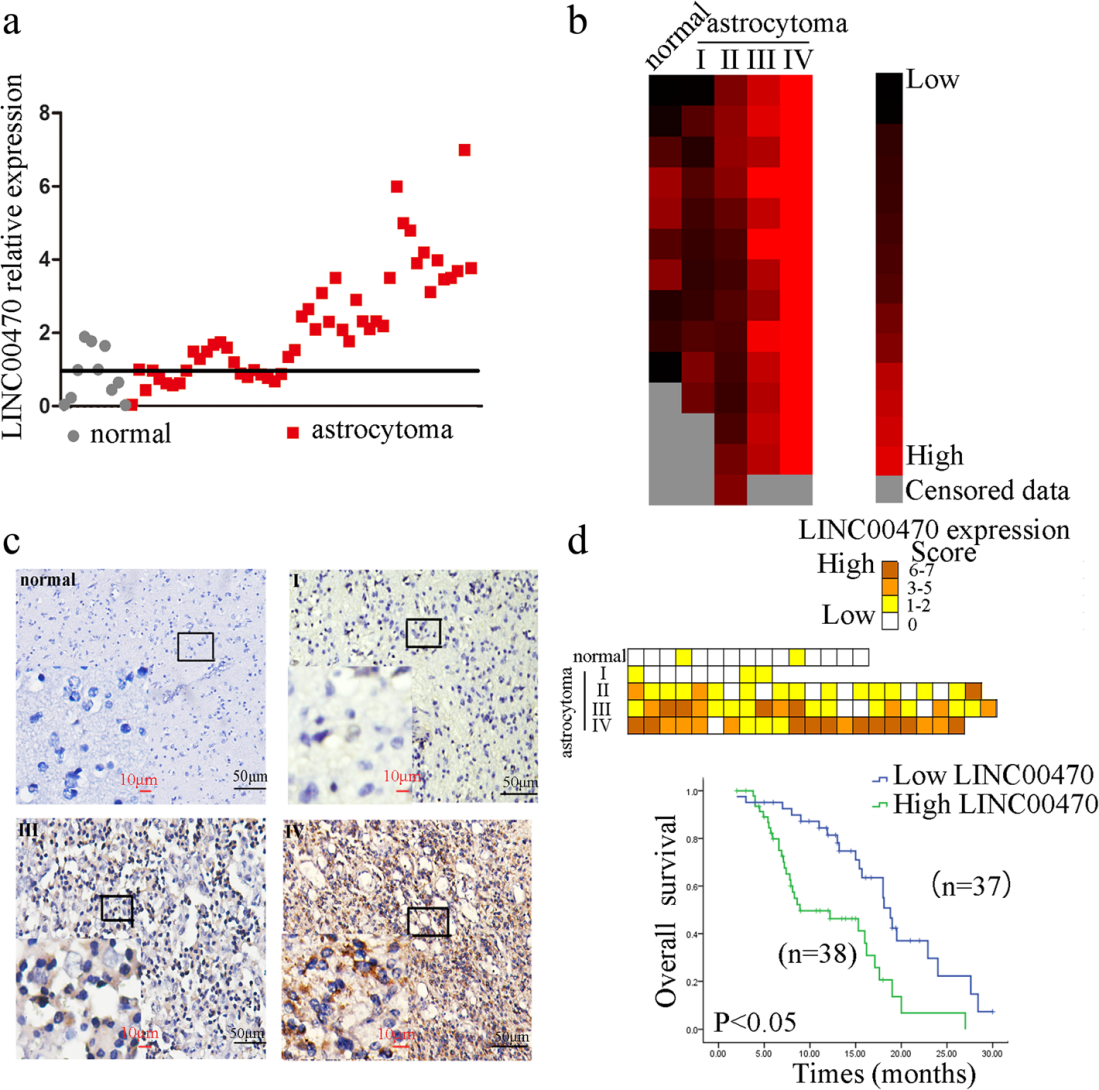
LINC00470 was an independent prognostic factor in astrocytoma patients. **a** RT-qPCR detected the expression levels of LINC00470 in normal brain tissues and astrocytoma. **b** RT-qPCR measured the expression levels of LINC00470 in astrocytoma with different WHO grades of astrocytoma. **c** The expression levels of LINC00470 were detected in astrocytoma tissues via in situ hybridization. Black scale bars, 50 μm; red scale bars, 10 μm. **d** Upper, the score of in situ hybridization in astrocytoma tissues; lower: Kaplan-Meier analysis for overall survival in 75 astrocytomas in high- and low-risk groups based on LINC00470 expression levels

To further evaluate the clinical significance of LINC00470 in astrocytomas, including GBMs, we found that the levels of LINC00470 were significantly increased in astrocytoma tissues (*n* = 60) compared with normal brain tissues (*n* = 12) by RT-qPCR (Fig. 7a), especially in high-grade astrocytomas (Fig. 7b). We next measured LINC00470 levels in a panel of 75 astrocytoma tissues and 15 normal brain tissues by in situ hybridization (Fig. 7c). The results were consistent with those of RT-qPCR (Fig. 7a).

Subsequently, we conducted a univariate cox regression analysis using clinical variables for astrocytoma patients and found that expression of LINC00470, astrocytoma grade, patients’ age, and the astrocytoma location were statistically associated with overall survival (Table 1). The multivariate cox proportional hazards model indicated that LINC00470 expression and astrocytoma grades were independently associated with overall survival (hazard ratio [HR] = 2.876, *P* = 0.02; HR = 1.892, *P* = 0.044; respectively) (Table 2). The results showed that LINC00470 was an independent prognostic factor in astrocytoma patients.

**Table 1:** Correlation between the clinicopathological factors and expression of LINC00470 in astrocytoma

Characteristic	Total(N=75)	LINC00470 high expression	LINC00470 low expression
Histologic grade^*^-no.(%)			
Astrocytoma			
I	9(12)	3(33)	6(67)
II	27(36)	14(52)	13(48)
III	18(24)	13(72)	15(28)
IV	21(28)	17(81)	4(19)
Sex-no.(%)			
Male	39(52)	17(44)	22(56)
Female	36(48)	21(58)	15(42)
Age^*^-no.(%)			
≤42	31(41)	10(33)	21(67)
>42	44(54)	35(80)	9(20)
Tumor location^*^-no./total no.(%)			
Frontal lobe	23/72(32)	9/23(39)	14/23(61)
Parietal lobe	19/72(26)	6/19(32)	13/19(68)
Temporal lobe	13/72(19)	10/13(77)	3/13(23)
Brainstem	3/72(4)	2/3(66)	1/3(34)
others	14/72(19)	7/17(50)	7/17(50)
Laterality-no./total no.(%)			
Left	32/72(44)	15/33(47)	17/33(53)
Right	25/72(35)	10/26(38)	16/26(62)
others	15/72(21)	7/16(43)	9/16(57)
Presenting symptom-no./total no.(%)			
Seizure	38/70(54)	21/38(55)	17/38(45)
Headache	12/70(17)	6/12(50)	6/12(50)
Sensory or visual change	9/70(13)	5/9 (56)	4/9(44)
Mental statue change	11/70(16)	6/11(55)	5/11(45)

Categorical distributions were compared with the use of Fisher’s exact test.

^*^P<0.01 for the difference among the molecular subtypes.

**Table 2:** Summary of multivariate analysis of Cox proportional hazards model for survival of patients with astrocytoma

Variable	Univarible Regression		Multivariable Regression	
	HR	P	HR	P
Gender(Female vs. Male)	1.23	0.244	1.150	0 .593
Age	1.46	0.332	1.398	0.475
Grade				
I+II vs. III + IV	1.741	0.051	1.892	0.044
Tumor location	1.021	0.871	1.566	0.111
Laterality	1.381	0.211	1.522	0.169
Presenting symptom	0.721	0.879	0.901	0.351
HighLINC00470 expression	2.113	0.030	2.876	0.021

The patients were divided into high or low LINC00470 expression groups according to the ISH scores. Kaplan-Meier analysis of the 75 patients with astrocytoma revealed that high LINC00470 expression levels significantly correlated with shorter survival times (Fig. 7d). High LINC00470 expression was significantly associated with a poor prognosis of astrocytoma patients.

## Discussion

Previous studies have shown multiple signaling pathways that are misregulated in human glioblastomas, such as RTK/PI3K/AKT/Foxos signaling pathway, p53, and Rb1 tumor suppressor pathways [48]. Given the complexity and redundancy of the signaling networks associated with glioma, targeting of critical oncogenic pathways might constitute a promising treatment approach [49]. For example, S109 treatment disturbed three pathways in glioma including the RTK/AKT/Foxos signaling pathway and the p53 and Rb1 tumor-suppressor pathways [48]. Although a multitude of studies have demonstrated the importance of PI3K in the activation of AKT, there have been reports suggesting that AKT activation can proceed in a manner that is independent of PI3K [2]. In the present study, we provided the evidence that LINC00470 was required for AKT cytoplasm activation and the interaction of LINC00470 and FUS was critical for AKT activation. Our results provided a new mechanism for AKT activity regulation, and we uncovered noncanonical AKT activation signaling by long non-coding RNA.

Recently, the study of lncRNAs has become important, with emerging evidence indicating that lncRNAs function as oncogenes and tumor suppressors, thus having an impact on one or more of the cancer hallmarks [50, 51]. The roles of a small number of lncRNAs such as HOTAIR, H19, and MALAT1 have been depicted in cancers, but little is known about LINC00470. Our study suggested an oncogenic role for LINC00470 in GBM. This was based on the following lines of evidence: (1) LINC00470 was upregulated in GBM and its expression was positively correlated with p-AKT; (2) ectopic expression of LINC00470 or knockdown of LINC00470 increased or suppressed AKT activity and tumor cell proliferation, respectively; and (3) re-expression of LINC00470 in LINC00470-KO cells was able to restore AKT activation.

FUS is a member of the Ewing’s sarcoma family of proteins that appears to translocate from the cytoplasm to the nucleus [52], and it is phosphorylated in response to radiotherapy [53]. However, to date, there is no evidence that lncRNAs can regulate FUS localization and activation. Our study demonstrated that FUS was a new binding partner of LINC00470. LINC00470 bound FUS, anchored it in the cytoplasm, and increased FUS expression in the cytoplasm to activate it. Our results not only revealed that FUS could be used as molecular scaffolding that bound LINC00470 and AKT but also upregulated phosphorylated AKT. In HEK293 cells with the absence of LINC00470, FUS was mainly located in the nucleus, and it transported AKT to the nucleus. However, in GBM cells, LINC00470 prevented FUS from transporting to the nucleus, so AKT was also activated and anchored in the cytoplasm. High levels of p-AKT decreased ubiquitination of HK1, so that the HK1 protein degradation rate was inhibited, and a higher level of HK1 affected glycolysis and inhibited cell autophagy. Our results further suggested that LINC00470 mediated AKT activation, at least in part, through interaction with FUS.

Finally, we confirmed the prognostic value of LINC00470 and that the high level expression of LINC00470 was an unfavorable prognosis marker for astrocytoma patients. Patients with high expression of LINC00470 had shorter survival times than those with low expression of LINC00470.

## Conclusions

**Fig. 8:**
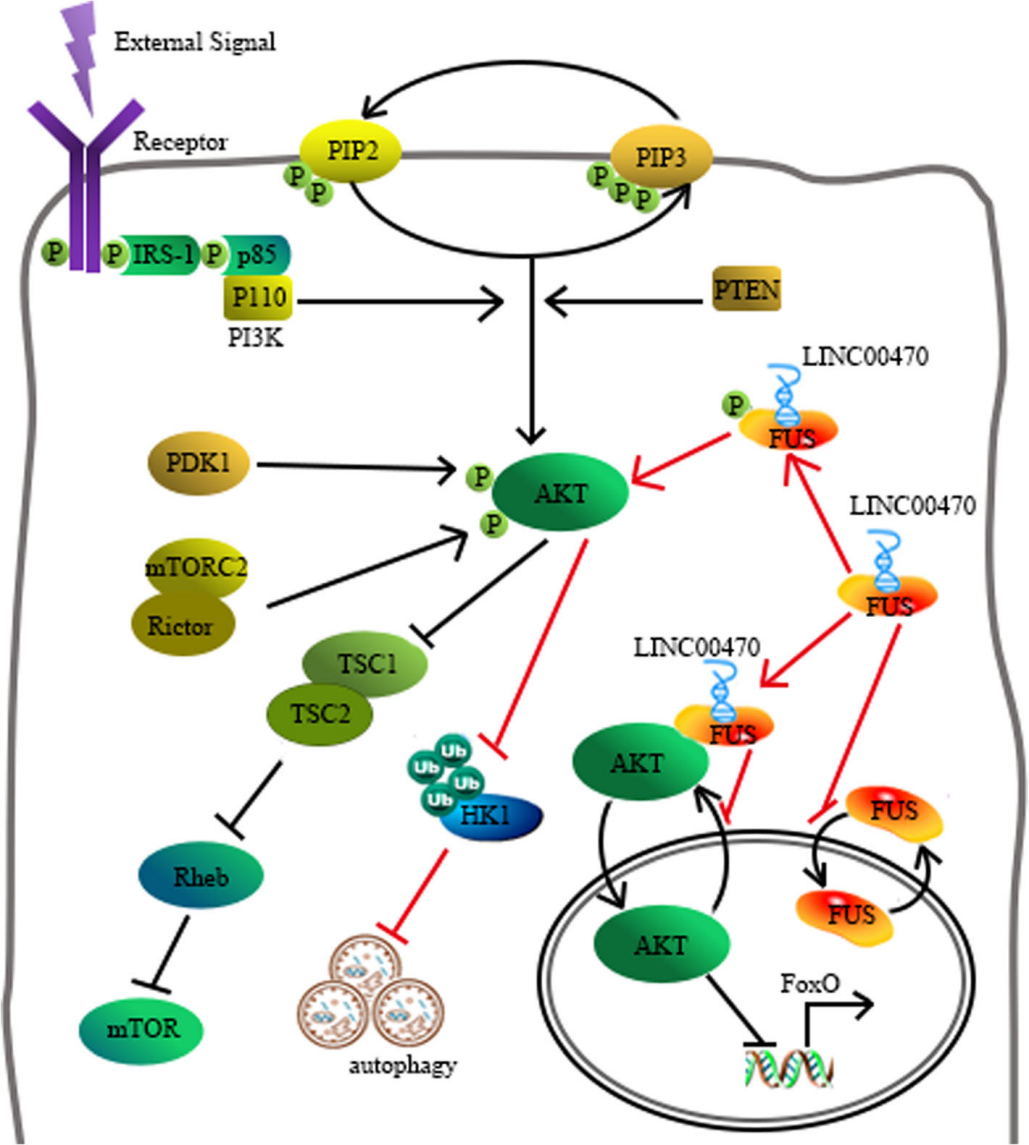
A schematic diagram of the working model for the LINC00470 targeting system in GBM cells

To summarize, we first demonstrated the function of LINC00470 in GBM and manifested a new regulatory mechanism for AKT activation. These results will provide a theoretical and experimental basis for verifying the mechanism of GBM carcinogenesis and identifying biomarkers for the early diagnosis and prognosis in GBM (Fig. 8).

## Additional files

Additional file 1: Bioinformatics analyses of evolutional conservation and protein-coding potential of LINC00470. A: the analysis of protein coding potential of LINC00470 using tools provided by the Peking University Center for Bioinformatics (cpc.cbi.pku.edu.cn/programs/run_cpc.jsp) shows LINC00470 lack of protein-coding capability. B: plasmids as schematically shown at left were transfected to HEK293 cells (right). Immunoblotting using antibody specific to ERK and fluorescent imaging showed that LINC0040-EGFP plasmid did not express GFP. (DOCX 755 kb)

Additional file 2: The relationship between LINC00470, AKT, and p-AKT. A: RT-qPCR and Western blotting measured the expression of LINC00470 and AKT in GBM cell lines and primary GBM cells. Data presented as mean ± S.E.M. of three independent experiments. B: Western blotting measured the expression of AKT and p-AKT in GBM cell lines and primary GBM cells. Data showed positive correlation between the expression of LINC00470 and p-AKT in GBM. (DOCX 302 kb)

Additional file 3: Effect of LINC00470 knockdown in GBM cells. RT-qPCR measured the expression of LINC00470 in GBM cell lines and primary GBM cells. Data presented as mean ± S.E.M. of three independent experiments. (DOCX 168 kb)

Additional file 4: The expression of PI3K in GBM cells. The expression of PI3K was measured by Western blotting in GBM cells. (DOCX 204 kb)

Additional file 5: The associate between LINC00470, FUS, and AKT in U87 cells. A: the interaction of LINC00470 and FUS was detected through RIP assays in U87 cells. Data are presented as the mean ± S.E.M. of three independent experiments. **p < 0.01. B: RNA pulldown showed binding between LINC00470 and FUS. Data are presented as the mean ± S.E.M. of three independent experiments. C: RIP assays showed that there was no interaction between LINC00470 and AKT in U87 cells. Data are presented as the mean ± S.E.M. of three independent experiments. (DOCX 264 kb)

Additional file 6: LINC00470, FUS, and AKT can form a ternary complex in U251 cells. The expression levels of LINC00470, AKT, and FUS were measured by RT-qPCR and Western blotting, respectively. Data presented as mean ± S.E.M. of three independent experiments. (DOCX 1262 kb)

Additional file 7: The expression levels of LINC00470 in GBM cells. The expression levels of LINC00470 were measured by RT-qPCR. Data presented as mean ± S.E.M. of three independent experiments. (DOCX 129 kb)

Additional file 8: LINC00470 promoted GBM cell proliferation. CCK8 assay was performed to determine the viability of primary GBM cells. Primary GBM cells were transfected with si-NC and si-LINC00470, pcDNA3.1, and pcDNA3.1-LINC00470, respectively.*p < 0.05, **p < 0.01. (DOCX 935 kb)

## Abbreviations

ANOVA: Analysis of variance
CCK-8: Cell counting kit-8
cDNA: Complementary deoxyribonucleic acid
Co-IP: Co-immunoprecipitation
GBM: Glioblastoma
GFP: Green fluorescent protein
LINC00470: Long intergenic non-protein coding RNA 470
RFP: Red fluorescent protein
RT-qPCR: Quantitative real-time polymerase chain reaction
siRNA: Small interference RNA
