# Supplementary material for: A cytoplasmic long noncoding RNA LINC00470 as a new AKT activator to mediate glioblastoma cell autophagy
Source: J Hematol Oncol. 2018 Jun 4;11:77. doi: 10.1186/s13045-018-0619-z (PMC5987392; doi:10.1186/s13045-018-0619-z)
Supplement: Supplementary file 1 — Bioinformatics analyses of evolutional conservation and protein-coding potential of LINC00470. A: the analysis of protein coding potential of LINC00470 using tools provided by the Peking University Center for Bioinformatics (cpc.cbi.pku.edu.cn/programs/run_cpc.jsp) shows LINC00470 lack of protein-coding capability. B: plasmids as schematically shown at left were transfected to HEK293 cells (right). Immunoblotting using antibody specific to ERK and fluorescent imaging showed that LINC0040-EGFP plasmid did not express GFP. (DOCX 755 kb) [file 13045_2018_619_MOESM1_ESM.docx]

**Additional file 1: Bioinformatics analyses of evolutional conservation and protein-coding potential of LINC00470**


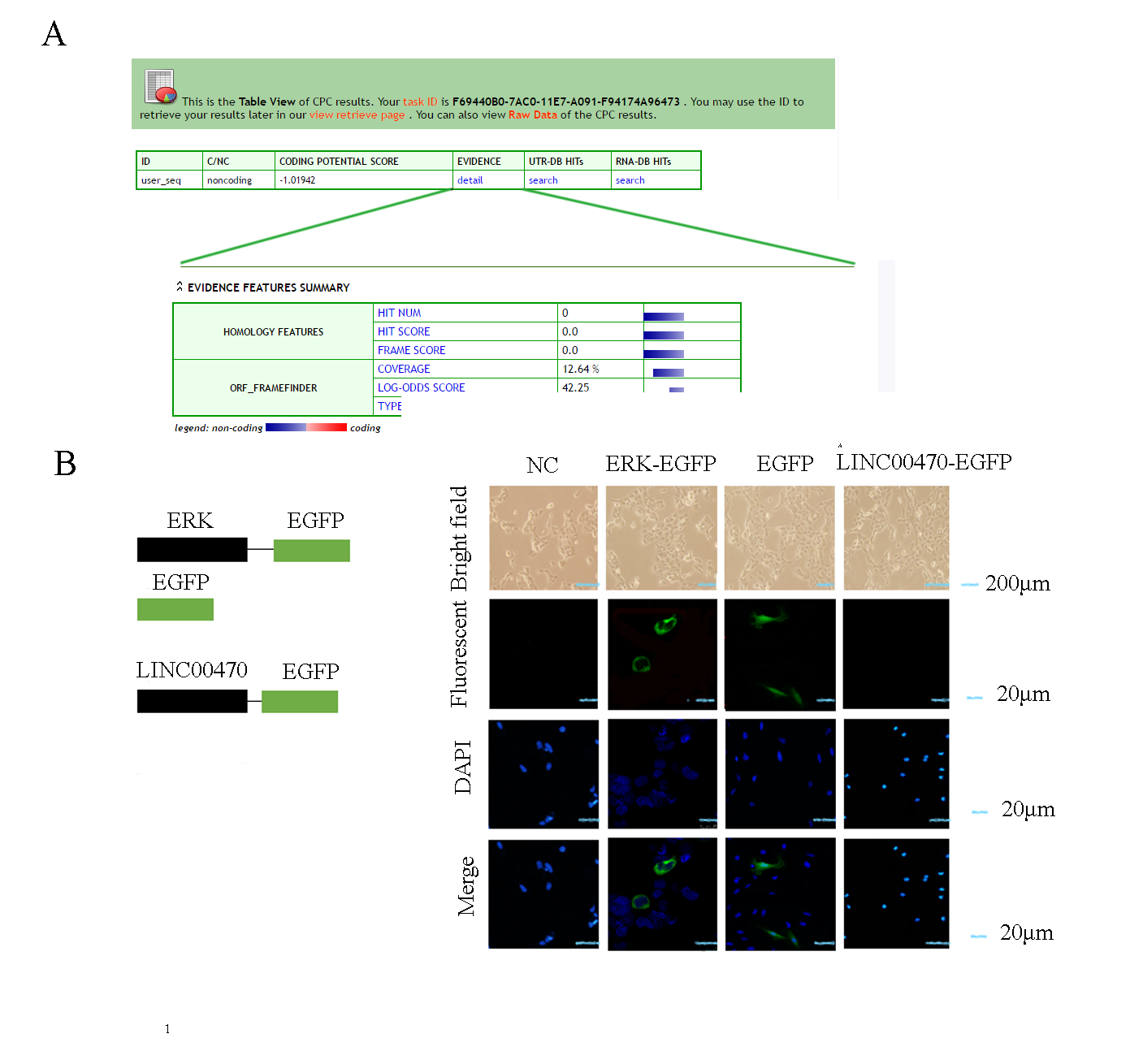


A: The analysis of proteincoding potential of LINC00470 using tools provided by the Peking University Center for Bioinformatics (cpc.cbi.pku.edu.cn/programs/run_cpc.jsp) shows LINC00470 lack of protein-coding capability.

B: Plasmids as schematically shown at left were transfected to HEK293 cells (Right). Immunoblotting using antibody specific to ERK and fluorescent imaging showed that LINC0040-EGFP plasmid plasmid did not express GFP.
