# Supplementary material for: A cytoplasmic long noncoding RNA LINC00470 as a new AKT activator to mediate glioblastoma cell autophagy
Source: J Hematol Oncol. 2018 Jun 4;11:77. doi: 10.1186/s13045-018-0619-z (PMC5987392; doi:10.1186/s13045-018-0619-z)
Supplement: Supplementary file 2 — The relationship between LINC00470, AKT, and p-AKT. A: RT-qPCR and Western blotting measured the expression of LINC00470 and AKT in GBM cell lines and primary GBM cells. Data presented as mean ± S.E.M. of three independent experiments. B: Western blotting measured the expression of AKT and p-AKT in GBM cell lines and primary GBM cells. Data showed positive correlation between the expression of LINC00470 and p-AKT in GBM. (DOCX 302 kb) [file 13045_2018_619_MOESM2_ESM.docx]

**Additional file 2 :****The relationship between LINC00470,AKT and p-AKT**


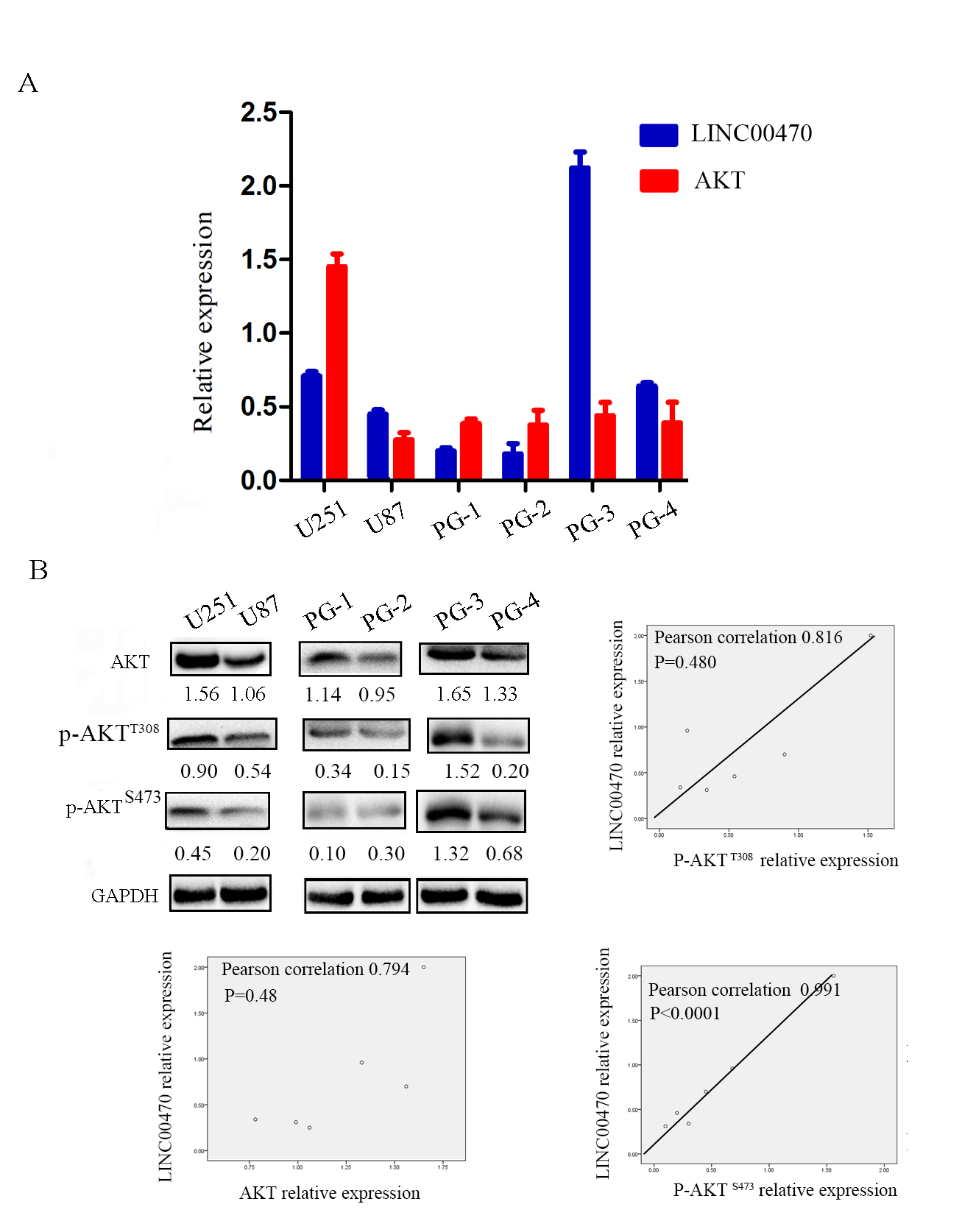


A: RT-qPCR and Western blotting measured the expression of LINC00470 and AKT in GBM cell lines and primary GBM cells. Data presented as mean±S.E.M. of three independent experiments.

B: Western blotting measured the expression of AKT and p-AKT in GBM cell lines and primary GBM cells. Data showed positive correlation between the expression of LINC00470 and p-AKT in GBM.
