# Supplementary material for: A cytoplasmic long noncoding RNA LINC00470 as a new AKT activator to mediate glioblastoma cell autophagy
Source: J Hematol Oncol. 2018 Jun 4;11:77. doi: 10.1186/s13045-018-0619-z (PMC5987392; doi:10.1186/s13045-018-0619-z)
Supplement: Supplementary file 4 — The expression of PI3K in GBM cells. The expression of PI3K was measured by Western blotting in GBM cells. (DOCX 204 kb) [file 13045_2018_619_MOESM4_ESM.docx]

**Additional file 4 :The expression of PI3K in GBM cells**


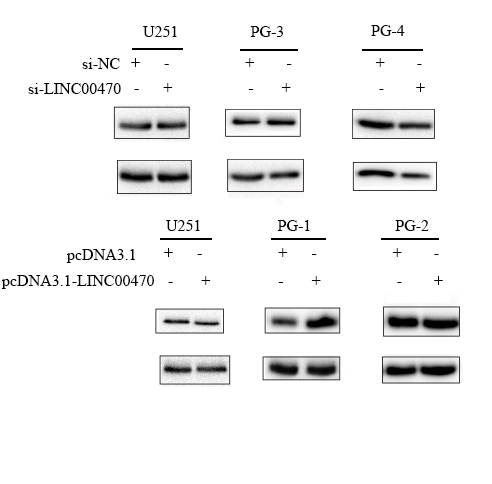


Expression of PI3K were measured by western blotting in GBM cells.
