# Supplementary material for: A cytoplasmic long noncoding RNA LINC00470 as a new AKT activator to mediate glioblastoma cell autophagy
Source: J Hematol Oncol. 2018 Jun 4;11:77. doi: 10.1186/s13045-018-0619-z (PMC5987392; doi:10.1186/s13045-018-0619-z)
Supplement: Supplementary file 5 — The associate between LINC00470, FUS, and AKT in U87 cells. A: the interaction of LINC00470 and FUS was detected through RIP assays in U87 cells. Data are presented as the mean ± S.E.M. of three independent experiments. **p < 0.01. B: RNA pulldown showed binding between LINC00470 and FUS. Data are presented as the mean ± S.E.M. of three independent experiments. C: RIP assays showed that there was no interaction between LINC00470 and AKT in U87 cells. Data are presented as the mean ± S.E.M. of three independent experiments. (DOCX 264 kb) [file 13045_2018_619_MOESM5_ESM.docx]

**Additional file 5 : The associate between LINC00470 ,FUS and AKT in U87 cells.**


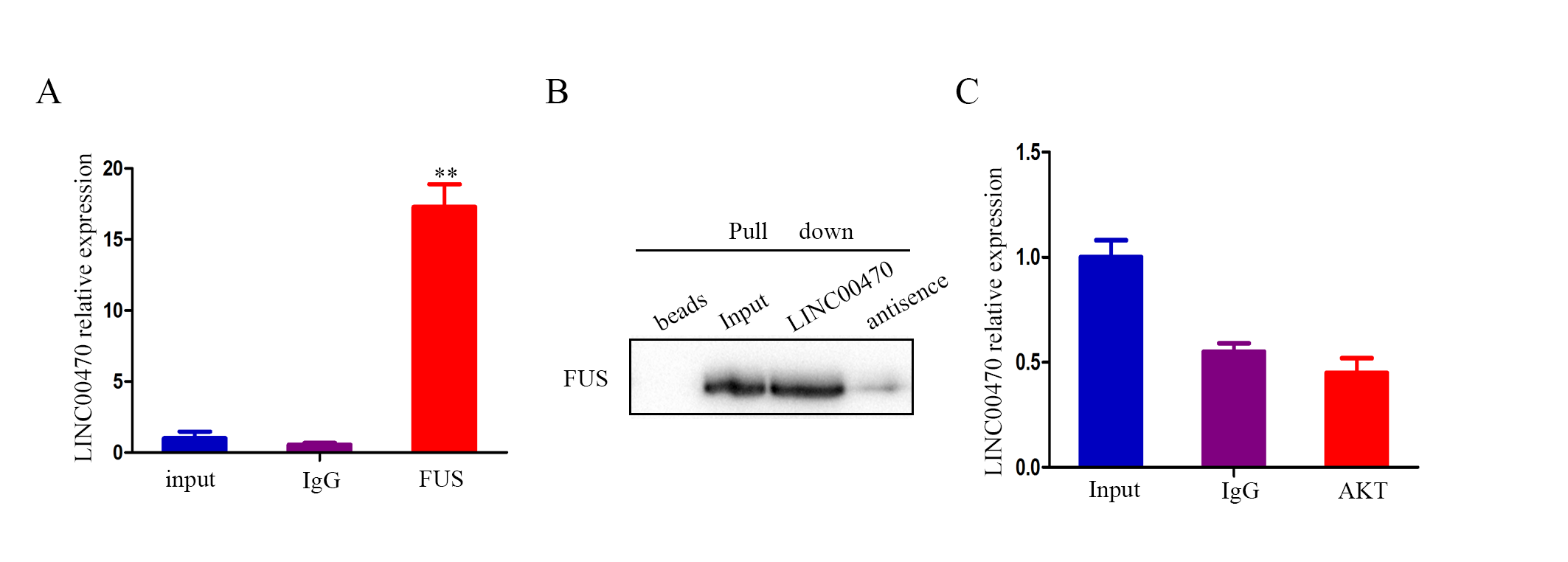


A: The interaction of LINC00470 and FUS was detected through RIP assays in U87 cells. Data are presented as the mean ± S.E.M. of three independent experiments. ***p* <0.01.

B: RNA pull-down showed binding between LINC00470 and FUS. Data are presented as the mean ± S.E.M. of three independent experiments.

C: RIP assays showed that there was no interaction between LINC00470 and AKT in U87 cells. Data are presented as the mean ± S.E.M. of three independent experiments.
