# Supplementary material for: A cytoplasmic long noncoding RNA LINC00470 as a new AKT activator to mediate glioblastoma cell autophagy
Source: J Hematol Oncol. 2018 Jun 4;11:77. doi: 10.1186/s13045-018-0619-z (PMC5987392; doi:10.1186/s13045-018-0619-z)
Supplement: Supplementary file 6 — LINC00470, FUS, and AKT can form a ternary complex in U251 cells. The expression levels of LINC00470, AKT, and FUS were measured by RT-qPCR and Western blotting, respectively. Data presented as mean ± S.E.M. of three independent experiments. (DOCX 1262 kb) [file 13045_2018_619_MOESM6_ESM.docx]

**Additional file 6:****LINC00470, FUS and AKT can form a ternary complex in U251 cells**


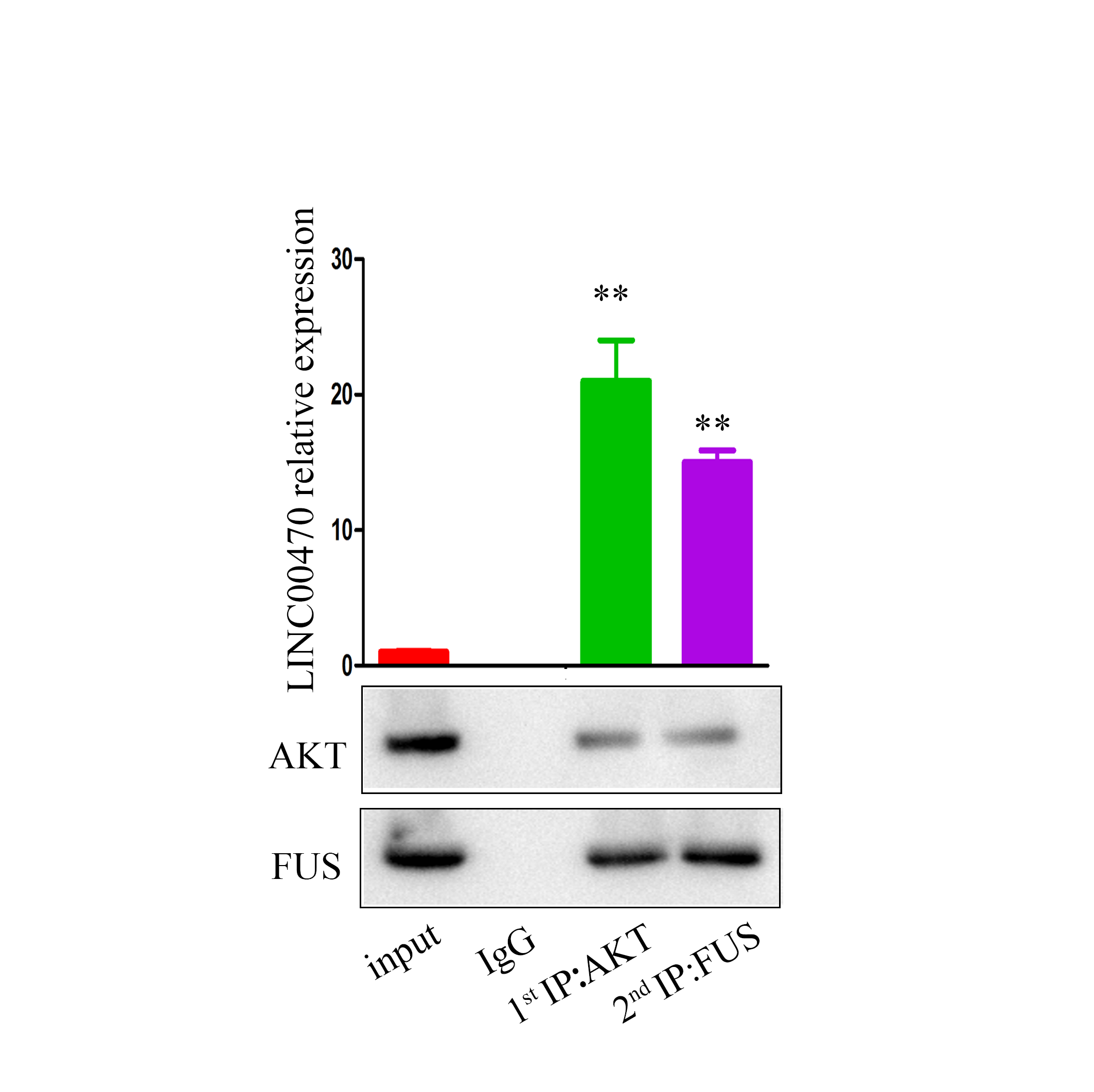


The expression levels of LINC00470, AKT and FUS were measured by RT-qPCR and western blotting, respectively. Data presented as mean±S.E.M. of three independent experiments.
