# Supplementary material for: A cytoplasmic long noncoding RNA LINC00470 as a new AKT activator to mediate glioblastoma cell autophagy
Source: J Hematol Oncol. 2018 Jun 4;11:77. doi: 10.1186/s13045-018-0619-z (PMC5987392; doi:10.1186/s13045-018-0619-z)
Supplement: Supplementary file 7 — The expression levels of LINC00470 in GBM cells. The expression levels of LINC00470 were measured by RT-qPCR. Data presented as mean ± S.E.M. of three independent experiments. (DOCX 129 kb) [file 13045_2018_619_MOESM7_ESM.docx]

**Additional file7:The expression levels of LINC00470 in GBM cells**


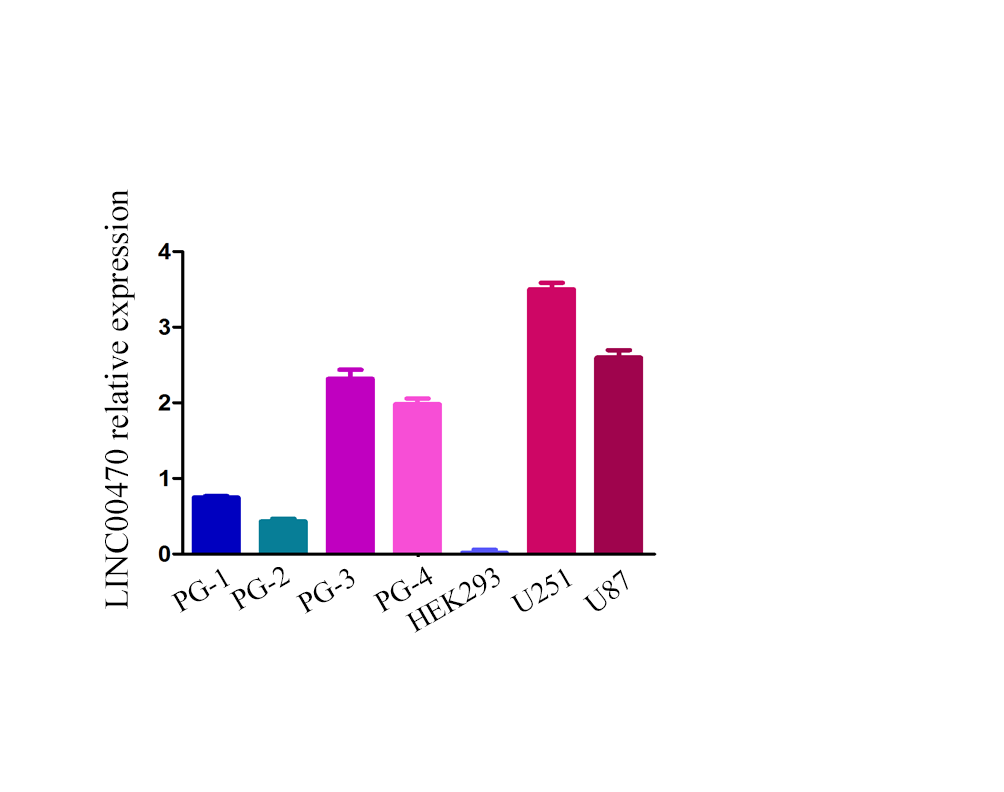


The expression levels of LINC00470 in GBM cells were measured by RT-qPCR.Data presented as mean±S.E.M. of three independent experiments.
