# Supplementary material for: A cytoplasmic long noncoding RNA LINC00470 as a new AKT activator to mediate glioblastoma cell autophagy
Source: J Hematol Oncol. 2018 Jun 4;11:77. doi: 10.1186/s13045-018-0619-z (PMC5987392; doi:10.1186/s13045-018-0619-z)
Supplement: Supplementary file 8 — LINC00470 promoted GBM cell proliferation. CCK8 assay was performed to determine the viability of primary GBM cells. Primary GBM cells were transfected with si-NC and si-LINC00470, pcDNA3.1, and pcDNA3.1-LINC00470, respectively.*p < 0.05, **p < 0.01. (DOCX 935 kb) [file 13045_2018_619_MOESM8_ESM.docx]

**Additional file 8: LINC00470 promoted GBM cell proliferation**


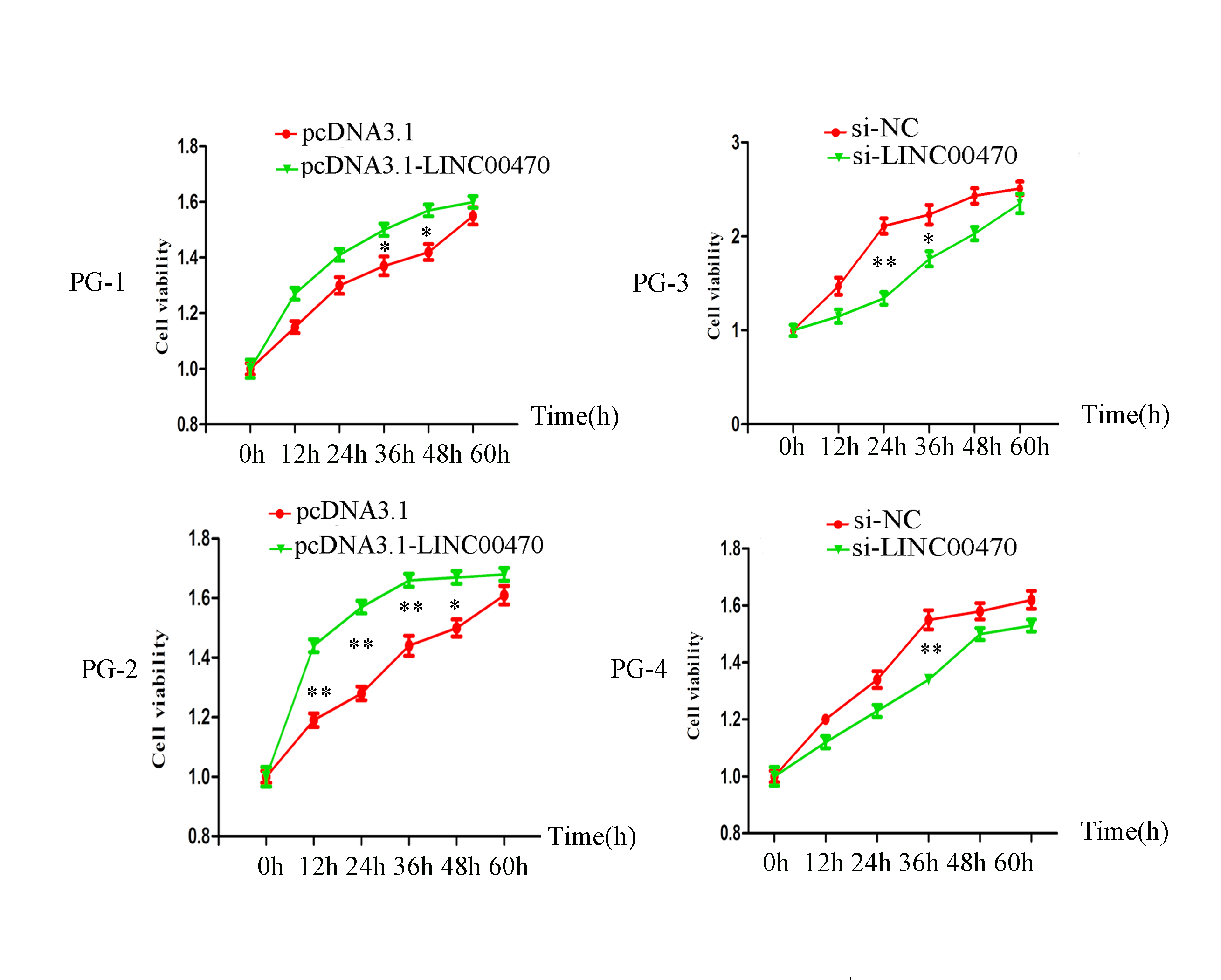


CCK8 assay was performed to determine the viability of primary GBM cells. Primary GBM cells were transfected with si-NC and si-LINC00470, pcDNA3.1 and pcDNA3.1-LINC00470, respectively.**p*<0.05,***p*<0.01
